# Breed differentiation in northern Ethiopian cattle: The application of univariate and multivariate analyses of phenotypic traits

**DOI:** 10.1371/journal.pone.0313190

**Published:** 2024-12-03

**Authors:** Amine Mustefa, Mulata Hayelom, Awoke Melak

**Affiliations:** 1 Animal Biodiversity Directorate, Ethiopian Biodiversity Institute, Addis Ababa, Ethiopia; 2 Mekelle Biodiversity Center, Ethiopian Biodiversity Institute, Mekelle, Ethiopia; Radford University Artis College of Science and Technology, UNITED STATES OF AMERICA

## Abstract

The aim of this study was to characterize and differentiate the Adwa, Arado, Medenes, and Begait cattle breeds in northern Ethiopia via univariate and multivariate analyses of phenotypic traits. Sixteen qualitative characteristics and nine morphometric traits were recorded for a total of 946 (604 females and 342 males) purposively selected adult cattle. The frequency, general linear model (GLM), and canonical discriminant (CANDISC) analysis procedures of the Statistical Analysis Software (SAS 9.0) were used to analyze the data. Qualitatively, Medenes and Begait, cattle breeds from lowland agroecology and livestock-based production systems, possess a convex facial profile, relatively larger dewlap, naval flap, and perpetual sheath, as well as more uniform and lighter body colors than the Adwa and Arado cattle breeds, which are from the midland agroecology and mixed crop–livestock production systems. Morphometrically, clear differences were observed among all of the studied cattle breeds, where the Begait, Medenes, Arado, and Adwa cattle breeds were ordered in descending order according to their overall body size. Moreover, multivariate analysis revealed significant differences among the breeds where each breed was placed under a separate group. However, such phenotypic distinctions among the four cattle breeds do not necessarily indicate genetic dissimilarities. Therefore, further inclusive genetic characterization studies involving the use of representative samples from the Adwa and Medenes cattle breeds are recommended to quantify the degree of genetic relationships among these breeds. Moreover, owing to the unknown breed-level population size, urgent conservation programs as well as genetic improvement strategies are needed to ensure sustainable utilization.

## Introduction

Ethiopia, a country in East Africa, has the largest cattle population on the continent [1], with 70.3 million cattle reported [2]. The reported cattle population makes cattle one of the most important livestock species that significantly contributes to the livelihood of individual farmers specifically and to the gross domestic product (GDP) of the country in general [2]. According to previous reports, cattle are farmed for milk, meat, and drought power, as well as for their manure [3–7].

Cattle are the most populous [2] and diverse [8, 9] livestock species in the country. Ethiopia is reported to have 28 registered cattle breeds [8]. However, some cattle breeds, including Adwa, Ambo, Bale, Hamer, Jemjem, Jigjiga, Medenes and Smada, have been registered without adequate phenotypic characterization studies [9]. Therefore, such gaps need to be addressed to provide a complete and country-wide picture of breed diversity and differentiation. This, in turn, helps us design breed-specific genetic improvement and conservation programs. Therefore, inclusive and exhaustive phenotypic characterization studies are needed to fill these gaps.

The current study targeted four DAD-IS-registered cattle breeds: the Adwa, Arado, Medenes, and Begait breeds. Adwa cattle were not phenotypically characterized but were registered in the country’s database on the basis of the assessment study report of Rege and Tawah [10]. The only available information about Adwa cattle is their distribution areas and some qualitative characteristics. Accordingly, Adwa cattle were reported to be distributed around the historic town of Adwa in the central zone of the Tigray region. They were reported to be predominantly red, chestnut, black, roan and white in color. Moreover, they have low milk production and are maintained mainly for their draught power [10].

The other cattle breed that is registered in the DAD-IS database without adequate phenotypic characterization studies is the Medenes cattle [9]. Medenes cattle are believed to be the result of the crossbreeding of Begait bulls and Arado cows [11, 12]. They were reported to be found in the western and northwestern zones of the Tigray region. [12] studied Medenes cattle by assessing only two morphometric traits (height at withers and heart girth) and a few characteristics of 25 males and 25 females. Accordingly, the Medenes cattle were medium-sized cattle with large ears and a dominantly black + white body color. Its medium body size, drought tolerance, and low milk production are the main parameters that distinguish Medenes cattle from Begait cattle [12].

Arado cattle are characterized by a uniformly patterned red body color, small body size and low milk production [4, 13]. Similarly, Begait cattle are characterized by a large body, black spots with a white background body color and relatively higher milk production than other indigenous cattle breeds [13–16].

Moreover, in addition to these phenotypic characterization studies, genetic characterization studies were also carried out to quantify the relationships of the Arado and Begait cattle breeds with other cattle breeds of Tigray and the adjacent Amhara and Afar regions [17–19]. However, comparisons were not made with the adjacent Adwa and Medenes cattle breeds. Therefore, the phenotypic relationships of both the Arado and Begait cattle breeds with the adjacent Adwa and Medenes cattle breeds remain unknown. Therefore, undertaking an inclusive phenotypic characterization study by taking a representative sample from each breed is mandatory to differentiate the cattle breeds as well as to quantify the degree of phenotypic relationships among these four cattle breeds of the Tigray region. Thus, the current study aimed to phenotypically characterize the Adwa, Arado, Medenes, and Begait cattle breeds and quantify their relationships.

## Materials and methods

### Ethics approval and consent of participants

The current study was approved by the Ethiopian Biodiversity Institute (EBI) from ethical and technical perspectives. The EBI is the primary institution responsible for the characterization, conservation and sustainable utilization of indigenous animal, plant and microbial genetic resources. Each participant farmer agreed to allow their animals to be measured using a centimeter-scale measuring tape. The measurements were carried out according to the [24] guidelines for the phenotypic characterization of farm animal genetic resources for food and agriculture. Additionally, permission regarding access to the field sites was obtained from the agricultural offices of the three districts: the Adwa district Agriculture Office, Tahtay Koraro district Agriculture Office, and Tahtay Adyabo district Agriculture Office.

### Study areas

The study was conducted in northern Ethiopia in the central and northwestern zones of the Tigray region. Three districts, i.e., the Adwa district of the central Tigray zone and the Tahtay Koraro and Tahtay Adyabo districts of the northwestern Tigray zone, were covered. Some parameters of the sampled districts, including weather conditions and agroecology, are presented in Table 1.

**Table 1:** Weather- and agroecology-related information of the selected districts.

Parameters	Districts		
	Adwa	Tahtay Koraro	Tahtay Adyabo
Agroecology	Midland–highland	Midland–highland	Lowland
Altitude of the district (m.a.s.l.)	1500–2700	1,500–2,300	800–1500
Altitude of the sampled locations (m.a.s.l.)	1876–2128	1920–1928	911–1212
Latitude	14°09’46”– 14°17’06” N	14°00’11”– 14°04’43” N	14°12’53”– 14°19’41” N
Longitude	38°53’37”– 39°00’56” E	38°22’10” –38°58’12” E	37°35’30” –37°53’16” E
Temperature (°C)	7.8–30.9	15–34	28–42
Rainfall (mm)	700–900	600–800	200–400
Area (km²)	635	640	3,838
Human population projection (2022)	113,438	78,483	108,743
Ethnicity	Tigrayans	Tigrayans	Tigrayans

[20–23].

#### Site and animal selection

Published and unpublished literature was reviewed alongside a quick preliminary assessment to identify the origin/breeding tract and distribution areas of each breed. Accordingly, representative samples of the Adwa, Arado, Medenes, and Begait cattle breeds were selected from their respective breeding tracts. Three districts were selected: the Adwa district of the central Tigray zone to represent the Adwa cattle breed [10], the Tahatay-Koraro district of the northwest Tigray zone to represent Arado cattle [4], and the Tahatay-Adyabo district to represent the Begait and Medenes cattle breeds [11–15].

Within each district, representative animals for each cattle breed were sampled from two purposively selected sites (***‘kebeles’***). Within each site (***‘kebeles’***), households that reared the targeted cattle breed were purposively selected. Finally, two unrelated adult cattle of the target breed, which were aged four years and above, were purposively selected from each household. The animal owners and trained laborers carefully controlled the animals during the measurement process. Aggressive animals that were unable to stand properly on flat ground were not considered.

### Data collection

The [24] guidelines for phenotypic characterization of farm animal genetic resources were used to collect both morphometric (quantitative linear body measurements) and morphological (qualitative characteristics) data. Data collection was carried out early in the morning to avoid errors associated with feeding and watering. Three researchers participated in the data collection procedure: two handled the quantitative data, while the remaining researcher handled the qualitative data. To reduce bias, morphometric data measurements were performed by the same researcher throughout the study. Animals were measured using a centimeter-unit textile measurement tape. Nine morphometric (Table 2) and 16 qualitative traits were recorded for a total of 946 cattle: 604 females and 342 males. According to [24], a 100–300 sample size is recommended for phenotypically characterizing a given breed. We sampled 210, 244, 250, and 242 Adwa, Arado, Medenes, and Begait cattle, respectively.

**Table 2:** Morphometric traits and their explanations.

No.	Morphometric traits	Definitions
1	Body length	Distance from shoulder point to pin bone
2	Heart girth	Chest circumference right behind its front two legs
3	Height at withers	Distance from ground to withers of the front foot
4	Pelvic width	Distance between the two ends of the pelvic bone
5	Muzzle circumference	Perimeter of the mouth
6	Ear length	Distance from the root to the tip of the back side of the ear
7	Horn length	Outer side distance between root and tip of the horn
8	Cannon bone length	Distance between the fetlock joint (ankle) and the knee
9	Hock circumference	Perimeter of the hock bone

Sources: [24, 25].

### Data analysis

The data were entered into the Microsoft Office Excel worksheet, and univariate and multivariate data analyses were performed via Statistical Analysis System (SAS) software 9.0 [26].

#### Univariate analysis

The normality of the data was tested via the univariate procedure of SAS. The qualitative and morphometric trait data were analyzed via the frequency (*chi-*square) and general linear model (GLM) procedures of SAS, respectively. The data were analyzed via the following model: Y_i_ = μ + X_i_ + e_i,_ where Y_i_ is an observation, μ is the overall mean, X_i_ is the fixed effect of breed (i = Adwa, Arado, Medenes, and Begait), and e_i_ is the random error. Means were compared via the adjusted Tukey‒Kramer test.

#### Multivariate analysis

Traits that better discriminated the cattle breeds were identified via stepwise discriminant analysis. Individual animals were assigned to known breeds via discriminant analysis. Canonical discriminant analysis was used to determine the maximal separation among the breeds as well as to plot the distances via the scored canonical variables. The pairwise distances between each breed were computed as $D^{2}(i|j)={\left(x_{i}-x_{j}\right)}^{\prime }cov^{-1}\left(x_{i}-x_{j}\right)$. Where *D*^2^(*i*\*j*) is the distance between breeds *i* and *j*, *cov*^-1^ is the inverse of the covariance matrix of measured variables, and *x*_*i*_ and *x*_*j*_ are the means of variables in the *i*^th^ and *j*^th^ breeds, respectively.

## Results

### Qualitative characteristics

**Fig 1:**
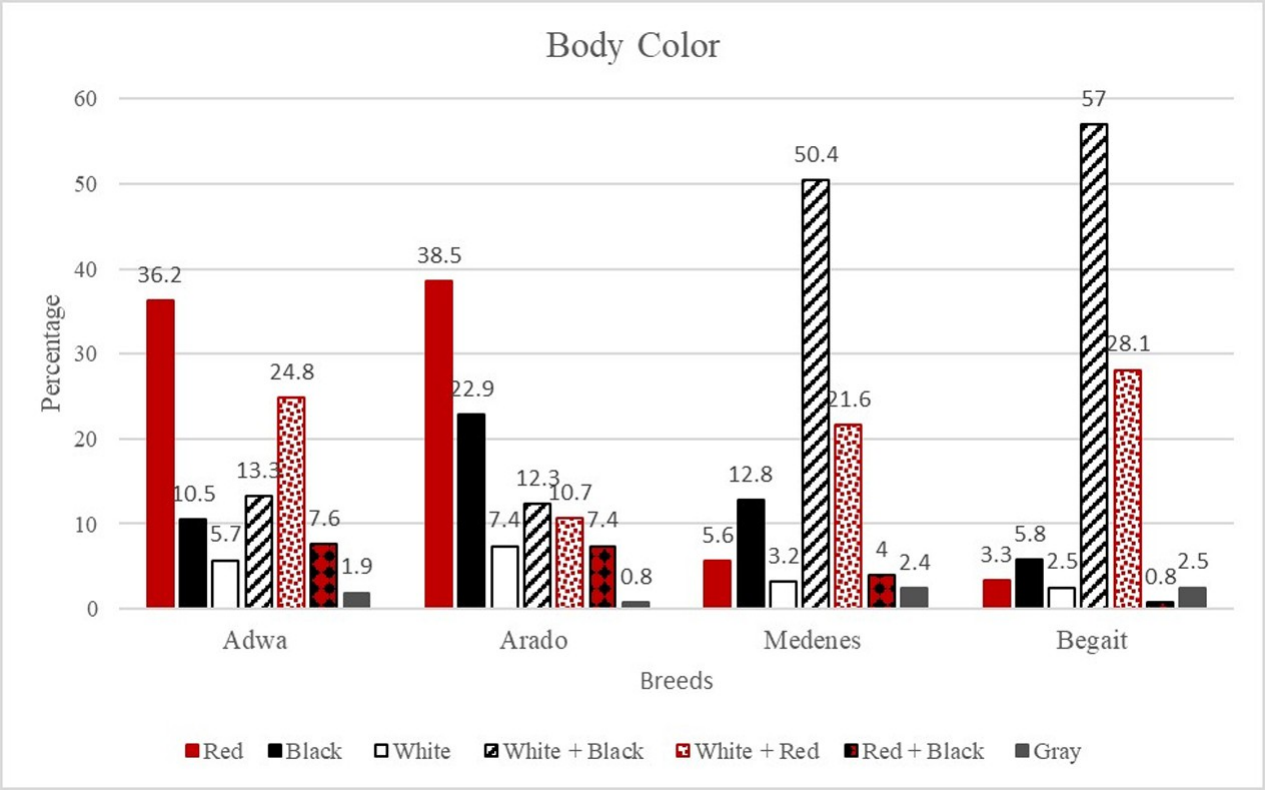
Body color of the four studied cattle breeds (chi-square value 159.2433, p<0.0001).

The body colors of the four studied cattle breeds are shown in Fig 1. Accordingly, body color was significantly affected by the breed of the animals. Overall, the results show some body color resemblances between the Adwa and Arado cattle breeds, where the majority of them possess a red body color. However, the frequency of black cattle was greater in Arado cattle than in Adwa cattle, whereas red + white cattle were observed more frequently in Adwa cattle than in Arado cattle.

Both the Medenes and Begait cattle breeds also possess comparable body colors, and the majority of these breeds exhibit a combination of black and white body colors. However, the frequency of red + white cattle was greater for Begait cattle than for Medenes cattle, whereas black cattle were observed more frequently for Medenes cattle than for Begait cattle.

The qualitative characteristics of the studied cattle breeds as well as their chi-square values and levels of significance are presented in Table 3. Ten out of the fifteen traits were significantly affected by the cattle breed. The overall results of the body color pattern are somewhat similar to the above body color results. Accordingly, the majority of the Adwa and Arado cattle breeds possess a uniform body color pattern. However, the proportion of uniform patterned cattle was greater in Arado cattle than in Adwa cattle. On the other hand, the proportion of spotted cattle was greater in Adwa cattle than in Arado cattle.

**Table 3:** Qualitative characteristics (in percentage values) of the studied cattle breeds.

Qualitative traits		Adwa	Arado	Medenes	Begait	*χ*^*2*^ value	*p*
Sample size		210	244	250	242		
Horn spacing	Narrow	14.3	10.7	20.0	17.4	4.5	NS
	Wide	85.7	89.3	80.0	82.6		
Horn shape	Straight	16.2	14.8	20.8	19.0	1.8	NS
	Curved	83.8	85.2	79.2	81.0		
Horn orientation	Lateral	8.6	10.6	16.0	18.2	13.5	NS
	Upright	22.8	23.8	28.8	23.9		
	Forward	67.6	63.1	53.6	52.9		
	Dropping	1.0	2.5	1.6	5.0		
Color pattern	Uniform	48.6	68.0	21.6	10.7	169.5	***
	Spotty	19.0	8.2	62.4	75.2		
	Pied	14.3	13.1	8.0	8.3		
	Shaded	18.1	10.7	8.0	5.8		
Hump shape	Erect	84.8	98.4	99.2	94.2	28.0	***
	Dropping	15.2	1.6	0.8	5.8		
Hump size	Small	31.4	50.0	36.0	27.3	38.6	***
	Medium	37.2	34.4	56.8	52.1		
	Large	31.4	15.6	7.2	20.6		
Hump position	Thoracic	30.5	29.5	8.0	22.3	22.5	***
	Cervico-thoracic	69.5	70.5	92.0	77.7		
Face profile	Straight	100	100	61.6	34.7	182.8	***
	Convex	0	0	38.4	65.3		
Back profile	Straight	97.1	98.4	88.8	86.8	17.	**
	Curved	2.9	1.6	11.2	13.2		
Tail length	Short	0	0.8	0	0	17.3	**
	Medium	1.9	5.8	0	0		
	Long	98.1	93.4	100	100		
Dewlap width	Small	1.0	2.5	2.4	0	89.4	***
	Medium	53.3	74.6	32.8	20.7		
	Large	45.7	22.9	64.8	79.3		
Naval flap width	Absent	41.5	28.2	3.1	0	146.5	***
	Small	41.5	51.3	27.1	10.7		
	Medium	11.3	12.8	27.1	14.7		
	Large	5.7	7.7	42.7	74.7		
Perpetual sheath	Absent	0	2.3	0	0	75.0	***
	Small	38.5	18.6	17.2	6.5		
	Medium	40.4	69.8	17.2	13.0		
	Large	21.1	9.3	65.6	80.4		
Ear shape	Straight edged	100	100	100	100	NA	NA
Rump profile	Sloppy	100	100	100	100	NA	NA

*χ*^*2*^: *Chi*-square, *p*: probability, NS: not significant, NA: not available

In line with the body color results, the Medenes and Begait cattle breeds also presented comparable body color patterns, with the majority exhibiting spotty color patterns. However, the spotty proportion was greater in Begait cattle than in Medenes cattle. On the other hand, the proportion of uniform patterned cattle was greater among the Medenes cattle than among the Begait cattle.

The majority of the Medenes and Begait cattle were medium humped, whereas the majority of the Arado cattle possessed small humps. On the other hand, comparable proportions of small and medium humped cattle were observed among the Adwa cattle. The majority of the studied cattle possessed humps at the cervicothoracic position, whereas the proportion of thoracic humped cattle was greater in Adwa and Arado. All of the Adwa and Arado cattle as well as the majority of the Medenes cattle had straight face profiles; however, the majority of the Begait cattle had convex face profiles.

The majority of the studied cattle possessed a straight back profile; however, a relatively greater proportion of cattle with a curved back profile were Begait and Medenes cattle. The majority of the studied cattle possessed long tails, whereas cattle with medium-length tails were also observed in the Adwa and Arado breeds. A greater proportion of cattle with large dewlaps, naval flaps, and perpetual sheaths were observed in Begait cattle than in Medenes cattle. The majority of the studied cattle possess widely spaced, forward-oriented, curved horns. Moreover, they possess erect humps, straight edged ears, and sloppy rumps.

### Morphometric traits

The morphometric measurements, least square means (LSM) together with standard errors (SE) and pairwise comparisons of the female and male cattle breeds are presented in Tables 4 and 5, respectively. All of the morphometric traits except the horn length of both the female and male cattle were significantly affected by their breed. Horn length measurements were comparable for all of the female cattle breeds. All of the female cattle breeds were significantly different from one another in all traits. The Adwa and Begait females had the lowest and the highest morphometric measurements, respectively. Moreover, the morphometric measurements of Arado females were significantly lower than those of Medenes female cattle.

**Table 4:** Morphometric measurements (LSM±SE) of the female cattle breeds.

Traits	Adwa	Arado	Medenes	Begait	*p*
Sample size	106	156	190	152	
Body length	99.5±0.66^d^	104.7±0.55^c^	116.1±0.51^b^	128.1±0.56^a^	***
Heart girth	133.2±0.85^d^	138.7±0.71^c^	154.2±0.65^b^	165.3±0.72^a^	***
Height at withers	102.1±0.65^d^	108.1±0.54^c^	119.3±0.50^b^	131.9±0.55^a^	***
Pelvic width	29.3±0.29^d^	31.1±0.24^c^	36.1±0.23^b^	38.5±0.25^a^	***
Muzzle circumference	32.7±0.26^d^	35.7±0.22^c^	38.2±0.20^b^	40.1±0.22^a^	***
Ear length	16.2±0.22^d^	19.1±0.19^c^	21.1±0.17^b^	22.5±0.19^a^	***
Horn length	20.7±0.99	21.3±0.83	23.7±0.77	23.0±0.84	NS
Cannon bone length	16.8±0.21^d^	19.5±0.17^c^	22.5±0.16^b^	25.7±0.18^a^	***
Hock circumference	25.3±0.25^d^	28.0±0.20^c^	32.5±0.19^b^	34.7±0.21^a^	***

*: p < 0.05

**: p < 0.01

***: p < 0.0001, NS: not significant.

**Table 5:** Morphometric measurements (LSM±SE) of the male cattle breeds.

Traits	Adwa	Arado	Medenes	Begait	*p*
Sample size	104	88	60	90	
Body length	104.9±0.79^d^	108.6±0.84^c^	118.1±1.02^b^	134.0±0.86^a^	***
Heart girth	144.5±1.12^c^	145.9±1.20^c^	155.6±1.46^b^	173.9±1.22^a^	***
Height at withers	108.5±0.83^d^	115.1±0.89^c^	122.5±1.08^b^	136.6±0.91^a^	***
Pelvic width	30.1±0.30^d^	31.9±0.32^c^	36.0±0.39^b^	38.2±0.33^a^	***
Muzzle circumference	35.8±0.34^d^	38.7±0.36^c^	40.4±0.44^b^	42.4±0.37^a^	***
Ear length	16.6±0.24^c^	20.3±0.26^b^	21.3±0.32^ab^	22.4±0.26^a^	***
Horn length	24.6±1.12	24.1±1.20	20.2±1.46	22.5±1.22	NS
Cannon bone length	17.4±0.22^d^	20.0±0.23^c^	22.8±0.28^b^	26.5±0.24^a^	***
Hock circumference	26.2±0.32^d^	30.3±0.35^c^	33.4±0.42^b^	36.6±0.35^a^	***

*: p < 0.05

**: p < 0.01

***: p < 0.0001, NS: not significant.

Similar to the results for the female cattle breeds, most of the male cattle were significantly different from one another in terms of all traits. Accordingly, Adwa and Begait males had the lowest and the highest morphometric measurements, respectively. Moreover, the morphometric measurements of the Arado males were significantly lower than those of the Medenes males. However, unlike females, Adwa and Arado males had comparable heart girth measurements. Similarly, compared with both the Arado and Begait males, the Medenes males had comparable ear length measurements, whereas the Arado and Begait males had significantly different ear length measurements.

### Multivariate analysis

#### Stepwise discriminant analysis

The morphometric traits entered in each step of the stepwise discriminant analysis when discriminating the cattle breeds are presented in Table 6. Accordingly, all nine morphometric traits were used to discriminate the four breeds within each sex. The height at the withers, hock circumference, and canon bone length within the females as well as the canon bone length, body length, and ear length within the males were the three most important morphometric variables used in discriminating the cattle breeds. Moreover, high partial R-square and F values, which are capable of differentiating the cattle breeds, were also observed.

**Table 6:** Order of traits used in discriminating the cattle populations from different breeds.

Sex	Step	Variables entered	Partial R-Square	F value	Pr > F	Wilks’ Lambda	Pr < Lambda
Females	1	Height at withers	0.8344	500.5	<0.0001	0.1656	<0.0001
	2	Hock circumference	0.2900	42.2	<0.0001	0.1161	<0.0001
	3	Canon bone length	0.1874	22.8	<0.0001	0.0943	<0.0001
	4	Body length	0.1623	19.1	<0.0001	0.0790	<0.0001
	5	Ear length	0.0939	10.2	<0.0001	0.0716	<0.0001
	6	Pelvic width	0.0601	6.3	0.0004	0.0673	<0.0001
	7	Muzzle circumference	0.0517	5.3	0.0014	0.0638	<0.0001
	8	Heart girth	0.0398	4.0	0.0079	0.0612	<0.0001
	9	Horn length	0.0188	1.9	0.1370	0.0601	<0.0001
Males	1	Canon bone length	0.8467	307.5	<0.0001	0.1533	<0.0001
	2	Body length	0.2863	22.2	<0.0001	0.1094	<0.0001
	3	Ear length	0.2739	20.8	<0.0001	0.0794	<0.0001
	4	Hock circumference	0.0934	5.6	0.0011	0.0720	<0.0001
	5	Heart girth	0.0867	5.2	0.0020	0.0657	<0.0001
	6	Pelvic width	0.0903	5.4	0.0015	0.0598	<0.0001
	7	Muzzle circumference	0.0880	5.2	0.0019	0.0546	<0.0001
	8	Horn length	0.0564	3.2	0.0253	0.0515	<0.0001
	9	Height at withers	0.0461	2.6	0.0569	0.0491	<0.0001

#### Discriminant analysis

The classification of each individual animal according to its corresponding breed together with the error rate for each breed under both sexes is presented in Table 7. Overall, the results show the highest percentages: 89.7% and 89.5% for females and males, respectively. The highest and lowest classifications into their respective breeds were observed in Medenes and Arado males, respectively. Medenes cattle were classified correctly, followed by Begait and Adwa cattle. The classification of Arado cattle was relatively lower than that of the other cattle.

**Table 7:** Number and percentage of observations classified into breeds.

Sex	From breed	Adwa	Arado	Medenes	Begait	Total
Females	Adwa	**94 (88.68)**	12 (11.32)	0	0	106 (100)
	Arado	16 (10.26)	**130 (83.33)**	10 (6.41)	0	156 (100)
	Medenes	0	6 (3.16)	**180 (94.74)**	4 (2.11)	190 (100)
	Begait	0	0	12 (7.89)	**140 (92.11)**	152 (100)
	Error rate	0.1132	0.1667	0.0526	0.0789	**0.1029**
Males	Adwa	**92 (88.46)**	10 (9.62)	2 (1.92)	0	104 (100)
	Arado	4 (4.55)	**68 (77.27)**	16 (18.18)	0	88 (100)
	Medenes	0	2 (3.33)	**58 (96.67)**	0	60 (100)
	Begait	0	0	4 (4.44)	**86 (95.56)**	90 (100)
	Error rate	0.1154	0.2273	0.0333	0.0444	**0.1051**

#### Canonical discriminant analysis

The results showing the canonical correlation, proportion, and eigenvalues of the three canonical structures (Can 1, Can 2, and Can 3) are shown in Table 8. Accordingly, the highest canonical correlation, proportion, and eigenvalues were obtained for the first canonical structure (Can 1) for both sexes. Can 1 had significant (above 1 Eigenvalue) potential for discriminating the studied cattle into separate groups. Alongside Can 1, Can 2 also had a significant eigenvalue, which is capable of discriminating the males into separate groups.

**Table 8:** Multivariate statistics outputs from the three canonical structures.

Multivariate Statistics	Females			Males		
	Can 1	Can 2	Can 3	Can 1	Can 2	Can 3
Canonical correlation	0.9540	0.4648	0.3834	0.9400	0.7115	0.3809
Proportion	0.9576	0.0261	0.0161	0.8641	0.1166	0.0193
Eigen value	10.12	0.28	0.17	7.60	1.03	0.17

Can = Canonical structure

The pairwise Mahalanobis distances between each breed for both sexes are shown in Table 9. Overall, each breed was distantly related, and each distance was found to be significant. Accordingly, each breed was found to be different and distinct from one another. The greatest distance was observed between Adwa and Begait females, whereas the distance between Arado and Medenes males was the shortest.

**Table 9:** Pairwise Mahalanobis distances between breeds. Females above diagonal, males below diagonal.

From District	Adwa	Arado	Medenes	Begait
Adwa	**0**	6.97 ***	32.67 ***	78.19 ***
Arado	8.16 ***	**0**	12.44 ***	44.76 ***
Medenes	21.48 ***	6.78 ***	**0**	11.83 ***
Begait	48.37 ***	30.31 ***	12.37 ***	**0**

**Fig 2:**
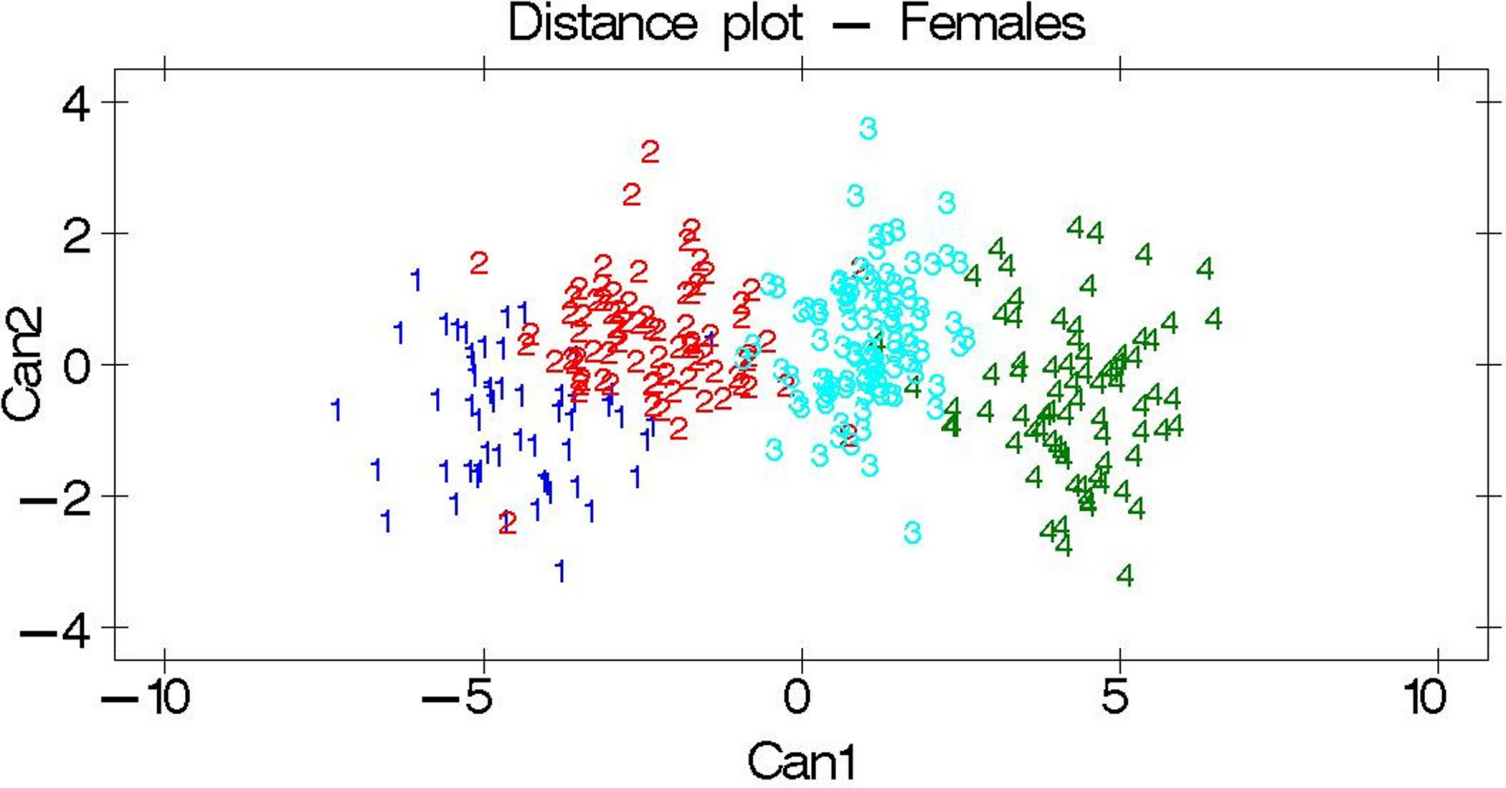
Distance plot among the females. Breeds are indicated by the numbers 1: Adwa, 2: Arado, 3: Medenes, and 4: Begait. Can 1 class means (Adwa = -4.54, Arado = -2.34, Medenes = 1.04, Begait = 4.28).

**Fig 3:**
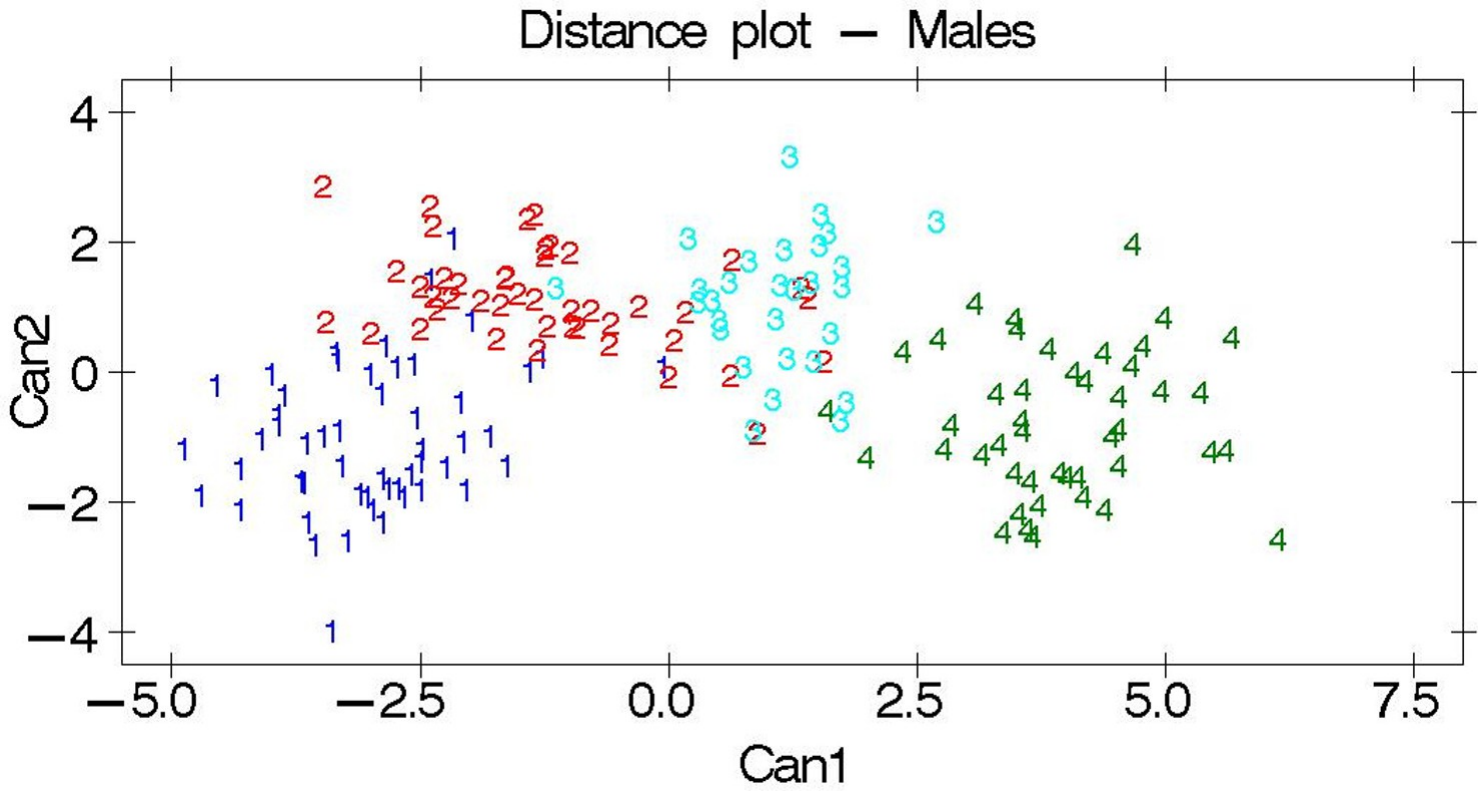
Distance plot among the males. Breeds are indicated by the numbers 1: Adwa, 2: Arado, 3: Medenes, and 4: Begait. Can 1 class means (Adwa = -3.00, Arado = -1.22, Medenes = 1.08, Begait = 3.94).

A distance plot of the first structure against the second canonical structure for both sexes is shown in Figs 2 and 3. In line with the results of the Mahalanobis distances, each studied cattle breed was found to be distinct and separate from one another.

## Discussions

### Qualitative characteristics

The qualitative characteristics of a given breed/s are among the outputs of phenotypic characterization, which can describe the breed/s easily because of their observable nature [24]. In addition to showing the outer characteristics of a given breed/s, qualitative traits can also be used to quantify the degree of relatedness among the breeds. According to the results shown in Fig 1 and Table 3, the qualitative characteristics of the Medenes and Begait cattle breeds were found to be comparable to the reports of [13–15] on Begait cattle as well as [12] on the Medenes and Begait cattle breeds. Similarly, the results for the Adwa and Arado cattle breeds were found to be comparable with those of previous reports [4, 13] for Arado cattle as well as [10] for Adwa cattle.

In line with the reports of [27] for Ogaden cattle, the cattle breeds from lowland agroecology, i.e., Medenes and Begait, possess more unique and lighter body colors than the cattle breeds from midland agroecology, i.e., Adwa and Arado. Moreover, the Medenes and Begait cattle breeds possess larger dewlaps, naval flaps, and perpetual sheaths than do the Adwa and Arado cattle breeds. These variations might be associated with their adaptation to their lowland production environment. On the other hand, in line with the reports of [28] for Harar cattle, the observed diverse and relatively darker body colors in the Adwa and Arado cattle breeds might be associated with the adaptation to the higher altitude of these cattle breeds than the Medenes and Begait cattle breeds. Animals with darker body colors are regarded as better at adapting to relatively highland and cold environments than are animals with lighter body colors because darker colors absorb more heat than lighter colors do [29].

The qualitative characteristics of the cattle breeds that shared similar agroecologies were similar. The Adwa and Arado cattle breeds showed some likeness in most of the studied qualitative characteristics, which might be due to the same midland-dominated agroecology and mixed crop-livestock-based production system shared by these two cattle breeds [4, 10]. Similarly, most of the qualitative characteristics of Medenes and Begait cattle were found to be comparable, possibly because these two cattle breeds share the same lowland-dominated agroecology and livestock-dominated production system [12, 13]. Moreover, this might be due to the dominance of the traits in the paternal line (Begait bull) over the traits in the maternal line (Arado cow) in making the Medenes cattle. In accordance with [12], the Medenes cattle breed was reported as crossbred between Begait bulls and Arado cows. According to [30], under a normal dominance situation, body color and other related qualitative characteristics of the crossbreed can be inherited from the parent with dominant traits.

Different breeds might share some common qualitative characteristics that might arise from shared similarities in their production environments [27, 28]. Ogaden cattle [28], Begaria cattle [7], and Nuer cattle [31] were reported to possess comparable qualitative characteristics, including plain patterned white body color and short horns. These cattle breeds share comparable lowland agroecology as well as pastoralist-type production systems. Similarly, three neighboring cattle breeds from the eastern lowland part of the country, i.e., Afar cattle [32], Kereyu cattle [33], and Raya cattle [34], were also reported to possess similar qualitative characteristics, including lyre-shaped long horns. Therefore, the currently observed likeness in qualitative characteristics alone might not lead to breed similarities. Thus, it is recommended to compare the overall body conformation and morphometric variations observed among the cattle breeds to quantify the overall degree of phenotypic relationship.

### Morphometric traits

In addition to showing the qualitative characteristics of a given breed/s, revealing their morphometric measurements is among the outputs of phenotypic characterization [24]. Moreover, in addition to their body measurements, morphometric traits can also be used to quantify the degree of resemblance among breeds. According to the results shown in Tables 4 and 5, morphometric traits easily separated the studied cattle breeds, where Begait, Medenes, Arado, and Adwa cattle breeds were ordered in descending order regarding overall body size. These results were comparable with the reports of [12, 13], who reported the body size dominance of Begait cattle over the other cattle breeds of the Tigray region, including Arado cattle.

Overall, the results indicated that the body size of the lowland cattle breeds, i.e., Medenes and Begait, was larger than that of the midland cattle breeds, i.e., Adwa and Arado, which was due to the need for a large body for heat dissipation in lowland areas [35]. According to [35], heat dissipation is greater in larger and taller animals than in smaller and shorter animals. Similar results have also been reported for Tigray sheep [36], short fat-tailed Merhabete sheep [37], Afar and Menz sheep [38, 39], and Sudanese and Egyptian desert goats [40].

According to [12], the Medenes cattle breed has a medium body size and was reported as a crossbred breed between the Begait and Arado cattle breeds. This was supported by the overall body size results of Medenes cattle from the current study, which lies between those of the Begait and Arado cattle breeds. Unlike qualitative traits, under normal dominance conditions, the morphometric measurements of a given crossbreed were expected to be between those of the two parent breeds [30].

In line with the reports of [9, 12–14], the overall morphometric measurements of Begait cattle from the current study make them the largest cattle breed in the country. The overall morphometric measurements of Arado cattle obtained from the current study were comparable to the values reported by [4] for Arado cattle in northwestern Tigray as well as [13] in the central and eastern Tigray region.

The Adwa cattle breed, the smallest cattle breed in the present study, was also smaller than most of the Ethiopian cattle breeds, i.e., Afar cattle [32], Arsi cattle [5], Begaria cattle [7], Fogera cattle [41], Gofa cattle [6], Gojjam Highland cattle [42], Harar cattle [28], Kereyu cattle [33], Nuer cattle [31], Ogaden cattle [28] and Raya cattle [34]. On the other hand, Adwa cattle had greater body length and height at the withers than did the Abergelle and Irob cattle, while their body sizes were comparable to those of Guraghe cattle [25] and Horro cattle [43].

The Adwa, Arado, Medenes, and Begait males are larger than their female counterparts are, which might be due to the effect of testosterone hormones secreted in males, which are responsible for muscle mass development [44]. On the other hand, the estrogen hormone, which is secreted by females, has a limited effect on their growth [44, 45]. The dominance of males over females was also reported by [46] in Mursi cattle [34], in Raya cattle [28], in Harar and Ogaden cattle breeds, and [25] in Guraghe cattle.

### Multivariate analysis

Multivariate analysis is capable of quantifying phenotypic relationships among breeds as well as discriminating phenotypically unrelated cattle breeds. The observed high partial R-square and F value outputs (Table 6) show the significance of the morphometric measurements for discriminating the studied cattle breeds. This was also supported by the observed low error rate outputs (Table 7), which can be interpreted as greater uniqueness and lower shared similarities among the breeds. This produces a greater chance of the breeds being categorized into different clusters as well as the uniqueness of each breed. Comparable outputs were reported by [28] for the phenotypically unrelated Ogaden and Harar cattle breeds.

The observed higher Eigenvalues (Table 8) for the canonical structures indicate that the separation among the studied breeds, with an Eigenvalue of 1 and above, was sufficient to separate breeds. Similarly, the greater differences between breed distances (Table 9 and Figs 2 and 3) quantify the phenotypic differences among the studied breeds. Accordingly, the Adwa, Arado, Medenes, and Begait cattle breeds were found to be phenotypically separate and different breeds from one another. However, such phenotypic results need to be supported by genetic characterization studies [47]. Accordingly, several molecular characterization studies have been carried out in the Tigray region; however, most of them did not consider the Adwa and Medenes cattle breeds [17–19]. Therefore, further genetic characterization studies involving the use of representative samples from the Adwa and Medenes cattle breeds are recommended.

#### Adwa cattle breed

Adwa cattle are found in the central Tigray zone around the Adwa, Ahferom and Werie districts. They possess diverse body colors and body color patterns: mainly a uniformly patterned red body color, white spots on a red background, and shaded red and white body colors. Adwa cattle are small compact animals with short body lengths and heights at their withers. The majority of Adwa cattle possess widely spaced, forward-oriented, curved horns as well as erected and medium-sized humps located at the cervicothoracic position. Moreover, they have a sloped rump and straight face and back profile. Furthermore, they possess straight edged ears, long tails, and medium–large dewlaps. The males possess small to medium-sized perpetual sheaths, whereas most of the females have either no or small naval flaps.

#### Arado cattle breed

Arado cattle are found in the northwestern, central, eastern, and southeastern Tigray zones. They possess diverse body colors; however, uniformly patterned red and black body colors are frequently observed. Arado cattle are small but relatively larger than Adwa cattle. The majority of Arado cattle possess widely spaced, forward-oriented, curved horns as well as erected and small humps located at the cervicothoracic position. Moreover, they have a sloped rump and straight face and back profile. Furthermore, they possess straight edged ears, long tails, and medium-sized dewlaps. The males possess medium-sized perpetual sheaths, whereas most of the females have small naval flaps.

#### Medenes cattle breed

Medenes cattle are found in the northwestern and western Tigray zones. They are medium-sized animals that are reported to be crossbreds of Begait bulls and Arado cows. The majority of them possessed relatively unique white colors with black spot body colors, while many white colors with red spots were also observed. The majority of Medenes cattle possess widely spaced, forward-oriented, curved horns as well as erected and medium-sized humps located at the cervicothoracic position. Moreover, they have a sloppy rump and straight back profile. A considerable number of Medenes cattle possess convex facial profiles, while the majority of them possess straight facial profiles. Furthermore, they possess straight edged ears, long tails, and large dewlaps. Males and females possess large perpetual sheaths and naval flaps, respectively.

#### Begait cattle breed

Begait cattle are found in the northwestern and western Tigray zones. They are the largest cattle in Ethiopia. The majority of them possessed relatively unique white colors with black spot body colors, while many white colors with red spots were also observed. The majority of Begait cattle possess widely spaced, forward-oriented, curved horns as well as erect and medium-sized humps located at the cervicothoracic position. Moreover, they have a sloppy rump and straight back profile. The majority of Begait cattle possess convex facial profiles, while a considerable number of Begait cattle with straight facial profiles were also observed. Furthermore, they possess straight edged ears, long tails, and large dewlaps. Males and females possess large perpetual sheaths and naval flaps, respectively.

## Conclusion

The Adwa, Arado, Medenes and Begait cattle breeds were phenotypically characterized at the farm level, and their phenotypic relationships were quantified via morphometric traits and qualitative characteristics. Similarities in qualitative characteristics were observed for cattle breeds that share similar agroecology and production systems. Accordingly, the Adwa and Arado cattle breeds were somewhat similar in most of the studied qualitative characteristics. Similarly, most of the qualitative characteristics of the Medenes and Begait cattle were found to be comparable. However, clear differences were observed in the morphometric traits among all of the studied cattle breeds. Moreover, multivariate analysis revealed significant differences among the breeds. Therefore, each of the cattle breeds was separated from one another. However, such phenotypic distinctions among the four cattle breeds do not necessarily indicate genetic dissimilarities. Therefore, further inclusive genetic characterization studies involving the use of representative samples from the Adwa and Medenes cattle breeds are recommended to quantify the degree of genetic relationships among these breeds. Moreover, the exact population size of each cattle breed is unknown, but the overall trend is reported to be decreasing for different reasons, including prolonged northern Ethiopian conflict and successive drought. Therefore, urgent conservation programs as well as genetic improvement strategies are needed to ensure sustainable utilization.
